# Physico-Chemical, Rheological, and Antiviral Properties of Poly(butylene succinate) Biocomposites with Terpene—Hydrophobized Montmorillonite

**DOI:** 10.3390/polym17222984

**Published:** 2025-11-10

**Authors:** Magdalena Zdanowicz, Mateusz Barczewski, Małgorzata Mizielińska, Piotr Miądlicki

**Affiliations:** 1 Center of Bioimmobilisation and Innovative Packaging Materials, Faculty of Food Sciences and Fisheries, West Pomeranian University of Technology in Szczecin, Janickiego 35, 71-270 Szczecin, Poland; mmizielinska@zut.edu.pl; 2 Polymer Processing Division, Institute of Materials Technology, Poznan University of Technology, Piotrowo 3, 61-138 Poznan, Poland; mateusz.barczewski@put.poznan.pl; 3 Engineering of Catalytic and Sorbent Materials Department, Faculty of Chemical Technology and Engineering, West Pomeranian University of Technology in Szczecin, Al. Piastow 45, 71-311 Szczecin, Poland; piotr.miadlicki@zut.edu.pl

**Keywords:** antiviral properties, barrier properties, biocomposites, montmorillonite, poly(butylene succinate), terpenes

## Abstract

The aim of the work was to obtain poly(butylene succinate)—a PBS biocomposite material with an addition of natural sodium montmorillonite (Na-MMT) modified with two selected terpenes: pinene (P) and limonene (L) or their mixture (PL)—and examine their physico-chemical, rheological, and antiviral properties. Na-MMT was effectively hydrophobized and intercalated (confirmed with FTIR, TGA, and XRD analysis results) with the terpenes via the solventless method. The materials were obtained via extrusion, and the films were formed using thermo-compression molding. The addition of the fillers slightly increased mechanical properties, but barrier properties towards oxygen and water vapor were significantly improved (OTR from 52 to 28 cm^3^/m^2^∙24 h and WVTR 21 to 11 g/m^2^∙24 h for PBS and composite, respectively) without alteration of polymer morphology (SEM, XRD, FTIR) or thermal and thermomechanical properties, despite high filler content (10 wt%) in the polymer matrix. Surface contact angle values of PBS/M, PBS/M-L, and PBS/M-PL exhibited antiviral properties and were tested using Φ6 bacteriophage. The composites can be used for materials in medical and food packaging applications.

## 1. Introduction

Interest in greener composites is increasing nowadays, due to serious environmental pollution, called “white pollution”, especially microplastics. Labeling many novel materials introduced to the market as “eco-friendly” and “bio-” may mislead users and consumers. Society has often assigned the “bio” prefix (e.g., biomaterials, bioplastic, biocomposite) to fully biodegradable or natural-based materials. However, many “biomaterials” are obtained from fossil fuels and are non-biodegradable as polyolefin-based composites with natural fibers or fillers (e.g., wood fibers, cellulose, or minerals), hybrid blends with non- and biodegradable polymers (polyolefins with thermoplastic starch). Additionally, many producers labeled products as suitable for recycling or containing some part of recyclate as “eco-friendly”, meaning those can be fully obtained from fossil-free sources. Moreover, many companies misuse “green adjectives”, leading to the greenwashing phenomenon. In 2023, the EU took action to address the phenomenon and formed the Proposal for a Directive on Green Claims [1]. The mentioned issues expose the necessity for designing and producing genuinely green materials, which can be obtained by developing biocomposites made with biodegradable matrix and natural, non-toxic fillers, fibers, or additives. The main applications of such biocomposites are food or medical packaging, so it is important to use safe components. Usually cellulose-derivatives, starch, or mineral/inorganic fillers (calcium carbonate, aluminosilicates, metal oxides, silica nanoparticles) [2] are used for the modification of biodegradable polymers such as poly-lactide (PLA), polyhydroxyalkanoates (PHAs), or poly(butylene succinate) (PBS). One of the biodegradable polymers that can also be obtained from fossil resources and bio-based monomers is PBS. This polyester synthesized from succinic acid and 1,4-butanediol exhibits high potential for the industry because of similar mechanical and processing properties to polypropylene (PP). Nonetheless, PBS is characterized by better barrier properties towards oxygen and aroma and a lower processing temperature range than its polyolefin counterpart [3].

The fillers usually increase the product surface area and improve the biocomposite barrier and selected mechanical properties [2]. Thanks to incorporating materials with specific chemical structures, additional functionality of the final composites, such as antimicrobial activity, was also reported [4,5,6,7]. Clays such as kaolin, zeolite, or montmorillonite are low-cost, abundant fillers that can be used for different polymers. Their chemical composition and crystallographic structure are directly related to a given area’s geographical latitude, past climate, and geological processes. Smectites usually form in humid and hot climates where hydrolysis occurs [8]. Montmorillonite (MMT) (from the smectite family) originates in the alteration of volcanic ashes induced by weathering. Their layers are arranged by an octahedral sheet of aluminum sheets between tetrahedral silicon sheets. The domination of oxide anions is attributed to the negative residual charge that is balanced by anions and a substitution of cations such as Si^4+^, Al^3+^, Fe^2+^, Fe^3+^, and Mg^2+^ with H^+^, Na^+^, and Ca^2+^ in the interface/gallery [2,8]. Hundreds or thousands of such aluminosilicate layers bonded with weak van der Waals forces form a particular MMT particle [9].

Montmorillonite (MMT) has been used as a thickening and thixotropic agent in the paint and mineral oil industry, an antifriction agent in the building industry, or an emulsion stabilizer in the pharmaceutical industry [10]. Due to the hydrophilic character of MMT, it is not compatible with more hydrophobic polymers; thus, organophilization is performed. This modification is based on the exchange of the interlayer cation into, e.g., organic quaternary compounds like ammonium or phosphonium salts, usually with alkyl chains leading to broadening the distance between clay platelets. This process not only increases the compatibility with the polymer matrix but also increases the space gallery, improving clay dispersion in the polymeric matrix [11]. We can divide the dispersion degree into two types: intercalation, when the space gallery is enhanced, and exfoliation, when the distance between layers is expanded from separate platelets into nano dimensions and the nanocomposites can be obtained. The organophilization of clay with quaternary salts is usually performed in the slurry, but in situ polymerization of liquid monomer with MMT presence can also lead to the formation of composite systems with an intercalated/exfoliated nanofiller [11,12]. Most organophilizing agents are synthetic and can be toxic to aquatic and terrestrial organisms. Moreover, they can increase the chance of co-selecting antibiotic-resistant bacteria [13]. These disadvantages make the researchers search for new solutions for inorganic clay functionalization. Bio-based compounds such as amino acids [14,15] and essential oils (EOs) [16,17,18,19,20] can be used for the modification of inorganic materials applied as polymeric composite fillers. EOs or their components—terpenes—can intercalate MMT and be adsorbed on the clay external layer [21,22,23,24,25], leading to their hydrophobization. Moreover, besides being strong antimicrobial and antifungal agents, essential oils and terpenes also exhibit antiviral properties [26,27,28,29]. The last COVID-19 pandemic case has shown that not only antibacterial but also antiviral materials for food or medical packaging are needed, where human hands transport viruses and can be adsorbed from aerosols. Two monoterpenes with antiviral properties [30,31] were selected for this study: pinene and limonene.

The aim of the work was to obtain green composites where all the used components were environmentally friendly. The novelties and advantages were as follows:

(i) Prepare biocomposites with biodegradable polymer as the matrix and natural mineral filler are modified with natural, active compounds.
(ii) Essential oils and terpenes are highly volatile, fragrant, and irritative and can cause corrosion of the processing machines and tools; therefore, the active compounds were introduced into the polymer matrix in the mineral carrier.
(iii) The clay was modified via a solvent-free method.
(iv) Due to terpenes exhibiting antiviral properties, also towards coronaviruses, antiviral properties of biocomposite films were investigated using bacteriophage Φ6 as a surrogate of SARS-CoV-2.

Before composite manufacturing, the applied MMT modification effects on the thermal and structural properties of potential PBS composite filler were analyzed using Thermal Gravimetry (TGA), Fourier Transform Infrared Spectroscopy (FTIR), and X-ray diffractometry (XRD). Mechanical properties of the biocomposites were evaluated with the static tensile test, while dynamic thermomechanical analysis (DMTA) supplemented with thermal characterization (Differential Scanning Calorimetry, TGA) allowed for a comprehensive assessment of the structure–property relationship related to interfacial changes differentiated by chemical modification of the applied filler. The morphology of the materials was characterized by XRD, FTIR, and Scanning Electron Microscopy (SEM) and supplemented with rheological behavior determination. Additionally, investigation of antioxidative properties was performed. In order to estimate the stake potential in the mentioned packaging sector, the barrier properties toward oxygen (oxygen transmission rate—OTR) and water vapor (water vapor transmission rate—WVTR) were measured instrumentally, and antiviral properties were studied using bioreactors.

## 2. Materials and Methods

### 2.1. Materials

Poly(butylene succinate) FZ91PM BioPBS™ (BioPBS, PTT MCC Biochem Co., Ltd., Rayong, Thailand) with a density of 1.26 g/cm^3^ and MFR (190 °C, 2.16 kg) was used as a polymer matrix. Sodium montmorillonite (M) (Cloisite Na^+^, density 2.86 g/cm^3^, dry particle size < 25 µm) was obtained from BYK-Chemie GmbH (Wesel, Germany). (-)-β-Pinene (P, 99%) and α-limonene (L, 99%) were purchased from Alfa Aesar. DPPH–(2,2-diphenyl-1-picrylhydrazyl radical) (Tokyo Chemical Industry Co., Tokyo, Japan) was used to investigate antioxidative properties. The analysis of the antiviral properties was conducted using bacteriophage DSM-21518 (as a SARS-CoV2 surrogate) and *Pseudomonas syringae* van Hall 1902 DSM 21482 (as Φ6 phage bacterial host) purchased from a collection from the Leibniz Institute Deutsche Sammlung von Mikroorganismen und Zellkulturen (DSMZ, Braunschweig, Germany).

### 2.2. Modification of Montmorillonite

The dried (115 °C, 4 h) aluminosilicate clay was modified according to the solventless method described in the work of Giannakas et al. [23], with a slight modification. A glass plate with terpene (16 g) was placed in the middle of a bigger glass plate filled with Na-MMT (20 g). The whole system was wrapped with aluminum foil and put into the dryer for 24 h at 120 °C (till liquid terpene evaporation).

### 2.3. Preparation of the Biocomposite Films

Before processing, PBS was dried overnight at 60 °C. Then, PBS with the fillers (native clay and modified) at a weight ratio of 90:10 was mixed mechanically to obtain a homogenous dispersion of the clay with PBS granulate and processed with a co-rotation twin-screw extruder with L/D = 40 (LabTech Engineering Co. Ltd., Samutprakarn, Thailand) with 10 heating zones at profile temperature 110/145/160/165 °C × 7 (80 rpm). Extrudates were pelletized after being cooled in forced airflow. In the next step, the melt-mixed composite pellets were compression-molded with a hydraulic press at 170 °C in a copper frame and cooled down under pressure (15 MPa). The thickness of the films was in the range of 0.19–0.23 mm. The extruded pellets and the films were stored in sealed PE bags in ambient conditions. Sample acronyms are PBS for unmodified polymer film and PBS/M-X for composites, where X is P, L, or PL, depending on the modifier.

### 2.4. Fourier Transformation Infrared Spectroscopy with Attenuated Total Reflection FTIR-ATR Analysis

FTIR-ATR spectroscopy (Perkin Elmer Spectrophotometer Spectrum 100, Waltham, MA, USA) was used to analyze the fillers as well as the polymeric films. The averages from 16 measurements of FTIR-spectra were taken in a wavenumber range of 4000–550 cm^−1^ and processed using Spectrum software (version 10.03.06.0100). 

### 2.5. X-Ray Diffraction (XRD) Analysis

XRD analysis was used to characterize the fillers (d-spacing of the dried clay and modified clay platelets) before their addition into the polyester matrix. The X-ray diffraction (XRD) patterns of the fillers were recorded with an Empyrean PANalytical (Malvern, UK) X-ray diffractometer using Cu K (λ = 0.154 nm) as the radiation source in the 2θ range 1–15° with a step size of 0.052. D-spacings were calculated using the Braggs Equation, as in Equation (1):

2dsinθ = nλ(1)

where λ is the wavelength of the x-ray, d is the spacing of the crystal layers (path difference), θ is the incident angle (the angle between the incident ray and the scatter plane), and n is the diffraction order.

### 2.6. Differential Scanning Calorimetry (DSC)

Studies of phase transitions were performed using Differential Scanning Calorimetry (DSC). Measurements were performed on PBS films sealed in hermetic aluminum pans using a differential scanning calorimeter (TA Instruments DSC Q2500 Discovery, New Castle, DE, USA) in the heating–cooling–heating cycles with the standard heating/cooling rate of 10 °C/min. The measurements were conducted in the temperature range of −50 to 250 °C under a nitrogen atmosphere. The glass transition temperature (Tg) values were taken as the onset point of the transition during the second heating stage. The melting temperature (Tm) and the crystallization temperature (Tc) were determined as the maximum of the endothermic (for melting) or exothermic (for crystallization) peaks of the diffractograms.

The determination of the oxidation onset temperature (OOT) was carried out in an oxidizing atmosphere at a constant heating rate of 10 °C/min and ranged from 20 to 250 °C. The measurements were performed in a Netzsch DSC 204 F1 Phoenix apparatus (Selb, Germany) and aluminum crucibles.

### 2.7. Dynamic Mechanical Thermal Analysis (DMTA)

The tests of viscoelastic properties of PBS films were carried out using Dynamic Mechanical Analyzer Q800 (TA Instruments, New Castle, DE, USA) with a film tension clamp at a frequency of 1 Hz, a heating rate of 3 °C/min, and a temperature range from −75 to 120 °C. The analysis was conducted twice for each material.

### 2.8. Rheological Behavior

PBS and its composite rheological properties were analyzed using an Anton Paar MCR 301 oscillatory rheometer (Graz, Austria) equipped with 25 mm diameter parallel plates and a 0.5 mm gap. The measurements were conducted at 140 °C. Preliminary tests were performed using the strain-sweep mode (10 rad/s) in the 0.01–100% range to ensure later experiments were located in the linear viscoelastic (LVE) region. Angular frequency-sweep measurements were performed at 5% strain in the 0.05 to 500 rad/s range.

### 2.9. Thermogravimetric Analysis (TGA)

The %wt content of the modifier in the clay as well as thermal stability of the PBS films were investigated using TGA Q500 (TA Instruments, USA). Tests of ca. 12–14 mg of the samples were performed on platinum pans under 25 mL/min airflow in the temperature range of 40–900 °C at a heating rate of 10 °C/min, in a nitrogen atmosphere for the clays (to calculate the content of the modifier) and in an air atmosphere for PBS-based samples.

### 2.10. Scanning Electron Microscopy (SEM)

The surface and cross-section of the PBS and its composites were examined using a scanning electron microscope (SEM). The films were placed on pin stubs and coated with a thin layer of gold in a sputter coater at room temperature (Quorum Technologies Q150R S, Laughton, East Sussex, UK). All micrographs have been taken using a Vega 3 LMU microscope (Tescan, Brno-Kohoutovice, Czech Republic). The SEM analysis was carried out using a tungsten filament with an accelerating voltage of 10 kV.

### 2.11. Testing of Mechanical Properties

The static tensile tests of the PBS films (without the additives and composites) were conducted with testing machine Zwick/Roell (Ulm, Germany) equipped with a 2.5 kN load cell on 10 mm width stripes. The grip separation was 25 mm and the testing speed was 100 mm/min. The test was carried out for at least 6 repetitions for each specimen at ambient conditions. Analysis of variance (ANOVA) with the post hoc Tukey test was used to compare the significant differences for each mean value (for all parameters). All analyses were performed assuming a significance level below 0.05.

### 2.12. Characterization of Barrier Properties

Barrier properties regarding oxygen (OTR) water vapor transmission rate (WVTR) were determined according to the previous work [32].

### 2.13. Contact Angle Measurement

The measurements of the surface’s contact angle were performed using an SEO contact analyzer, Phoenix-Mini (PM-041807, Suwon, Republic of Korea), and Surfaceware 8 software after distilled water drop deposition in ambient conditions.

### 2.14. Antioxidative Properties

Antioxidative properties were investigated using 2,2-diphenyl-1-picrylhydrazyl radical DPPH measuring free radical scavenging capacity as is in previous work [33]. The methodology, results, and discussion are presented in the Supplementary Information (SI).

### 2.15. Antiviral Tests

To investigate antiviral properties/activity of the PBS and its composites, the Φ6 bacteriophage was prepared according to Bhetwal et al.’s [34] and Bonilla et al.’s [35] reports. As a phage host, *Pseudomonas syringae* was used. To evaluate the host’s growth rate in real-time, after its 24 h contact/cultivation with the PBS and PBS composites, Φ6 particles were kept in contact (incubated) with the films (each PBS or PBS composite film separately) according to the ISO 22196-2011 [36] standard. Simultaneously, *P. syringae* was incubated in LB broth overnight at 28 °C. Then, 1 mL aliquots of the bacterial suspension obtained after incubation (10^8^ CFU/mL) were mixed with 4 mL of the LB top agar to prepare inoculums. Next, the inoculums were poured onto LB agar plates, and they were left until the agar had gelled (~5 min). Then, serial dilutions (10^−1^–10^−8^) of the Φ6 lysates (after their incubation with PBS film and its composites) were used to determine phage titer reduction. Then, 20 μL of each diluted lysate, as well as of the undiluted lysate, were introduced into each of the nine sectors previously marked on the bottom of the plate. The plates were incubated at 28 °C for 24 h. After incubation, the plaques were counted and phage titer was calculated (phage titer was determined in plaque-forming units, PFU/mL, over six repetitions). Statistical significance was evaluated through analysis of variance (one-way ANOVA). The number of treatments (n) was 6. All analyses were evaluated with GraphPad Prism 8 (GraphPad Software, San Diego, CA, USA). To evaluate the host’s growth rate in real-time, the *P. syringae* overnight inoculum/culture was added to 10 mL of LB broth and cultivated at 28 °C until OD = 0.2 (optical density, in the BioSan bioreactors, BS-010160-A04, BioSan, Riga, Latvia). Finally, 10 µL of each phage lysate was poured into the 10 mL of the host culture (when OD was 0.2, MOI = 1, 1 phage per 1 bacterial cell). Five Φ6 lysates were amplified in the bacterial cultures (1 lysate sample—after its incubation with the PBS, control sample of its titer was 1.29 × 10^7^ [PFU/mL], and there were 4 lysate samples after their individual incubation with the PBS composites). *P. syringae* cultures containing Φ6 particles were incubated for 24 h at 28 °C. Five experiments were performed simultaneously.

## 3. Results

### 3.1. Characterization of the Fillers

#### 3.1.1. TGA of the Fillers

Table 1 shows the results of the determination of the modifier content in the clay using TGA. The modification was performed with the same amount of terpenes in the same condition. However, the clay adsorbed pinene at the highest amount (ca. 14%). It might be related to the higher volatility of pinene than limonene. The lowest amount (ca. 9%) was obtained for the terpenes’ mixture. The thermograms are presented in the Supplementary Information (SI), Figure S1. It can be observed that in modified MMT the curves have three steps of weight loss. The first step is related to the evaporation of terpenes absorbed more freely on the clay surface and residual moisture molecules. The second peak is related to the gradual release of terpene molecules “trapped” between clay platelets. Compared with pure terpenes that are highly volatile, the maximums of DTG peaks are 295, 307, and 302 °C for M-P, M-L, and M-PL, respectively.

#### 3.1.2. Characterization of the Fillers Using FTIR-ATR

**Figure 1:**
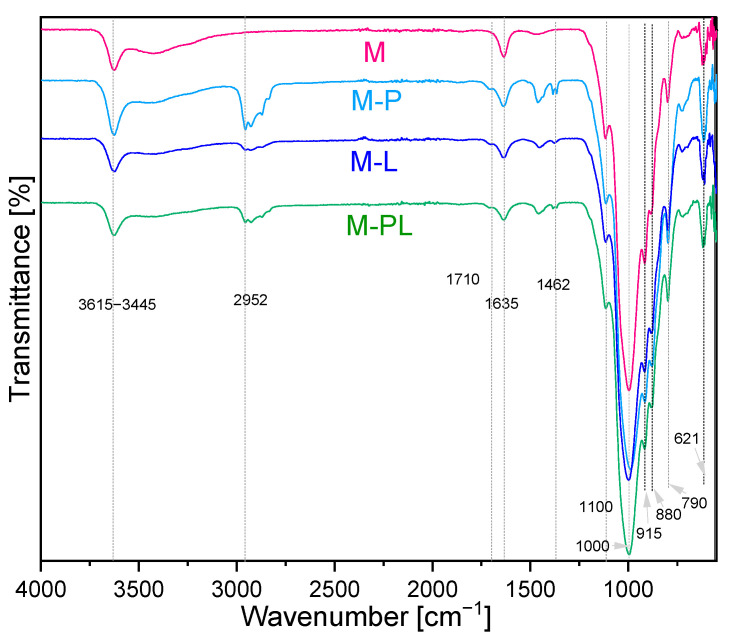
FTIR−ATR spectra for sodium montmorillonite (M) and modified clay with terpenes and their mixture.

Figure 1 shows FTIR-ATR spectra of pristine sodium montmorillonite and its derivatives. The bands at 3615 cm^−1^ and 3445 cm^−1^ for all the samples are attributed to stretching vibrations, while the band at 1635 cm^−1^ is assigned to bending vibrations of -OH groups from water molecules [37]. The bands corresponding to the AlAlOH and AlMgOH bending vibrations are observed at 915 and 880 cm^−1^, respectively. A broad band at 1000 cm^−1^ is related to the stretching vibrations of the Si-O groups, while the bands at 790 cm^−1^ and 1100 cm^−1^ belong to asymmetric stretching vibrations or bending vibrations of Si-O-Si bonds [38,39]. The band at 621 cm^−1^ is assigned to the Al-O and Si-O couple from the plane vibrations [40]. It can be observed that the clay, after modification, differs from the pristine MMT. Spectra of the modified clays reveal additional absorption bands, absent from the pristine M, originating from the terpenes, confirming their presence in the aluminosilicate. The peaks at ca. 2952 cm^−1^ are assigned to -CH_3_ groups of pinene and limonene, and a small peak at 1462 cm^−1^ is assigned to the vibrations of the skeletal C-H bond of these groups. Additionally, geminal methyl groups (1384 and 1366 cm^−1^) [41] can be noticed in samples with pinene. Peaks characterized for pinene in the M-P sample are clearly visible, which can indicate that terpene was also adsorbed at the MMT surface in a greater amount than limonene.

Moreover, for this series, there is a shift in the peak assigned to the stretching vibration of Si-O at 991 cm^−1^ [42] for native MMT to the lower wavenumber (983 cm^−1^), whereas for other samples, the maximum of the peak is at a higher wavenumber (M-L 995 cm^−1^ and M-PL 1001 cm^−1^). For modified clays, there are small, barely visible absorption bands at 1710 cm^−1^ that can correspond to carbonyl groups of derivatives of terpenes [43]. Bentonite is known as a catalyst (acting as a Lewis or Brönstead acid), which may oxidize and/or isomerize limonene; the oxidation products exceed the isomerized, especially when the reaction is prolonged [44]. Limonene can oxidize to carveol, carvone, and limonene oxide and pinene to verbenone [43,45]. 

#### 3.1.3. Analysis of the Fillers with XRD

**Figure 2:**
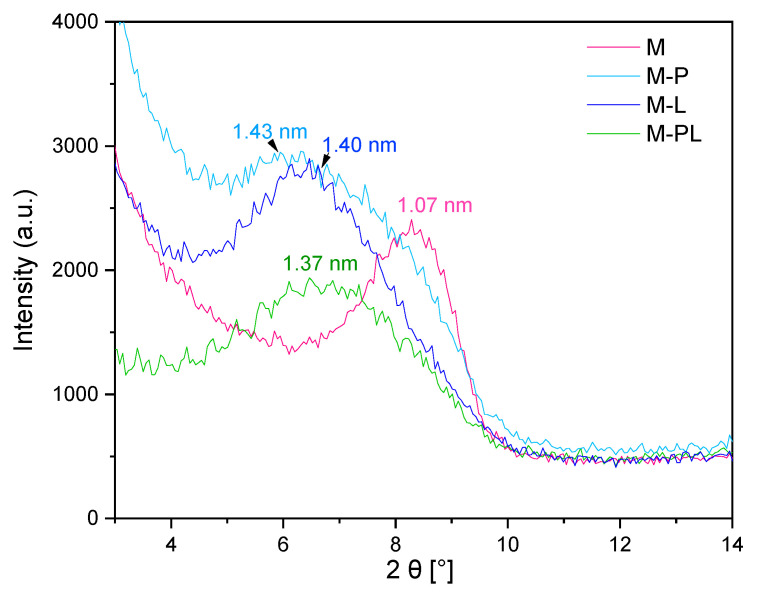
XRD of the montmorillonites.

We can observe shifts in peaks for modified MMT toward lower angle values, indicating an increase in the aluminosilicate interlayer gallery (d-spacing) after treatment of the filler with the selected terpenes or their mixture (Figure 2). D-spacing of pristine clay is 1.07 nm (8.3°), and MMT with terpenes is in the range of 1.31–1.43 nm. The biggest shift was recorded for M-P. Although this phenomenon correlates with TGA results (Table 1) (the highest content of pinene in MMT), there is no significant difference between the two terpenes, despite the fact that P content is twice higher than L (Table 1). It may be related to an adsorption of some P molecules also on the clay surface. The smallest shift towards lower angle values was observed for M-PL. It can be related to the more complex structure of the mixture than the individual terpenes, which can lower its volatility and hinder the sorption between the MMT layers. Broadening of the space gallery of Na-MMT depends on the modification method and chemical composition of the modifier. Pure terpenes can broaden the space gallery more effectively than their mixtures in the form of essential oils via the solventless method, e.g., in work [23], thyme, oregano, and basil EOs did not intercalate into Na-MMT (they adsorbed on the clay surface), and more effective intercalation was for already organophylized (with quaternary compounds) MMT [23,46]. Pure eugenol broadened the space gallery of MMT from 1.08 to 1.43 nm via the adsorption method [20].

### 3.2. Characterization of the PBS Films

There is no difference in chemical morphology between PBS and composites. Results are presented in SI.

#### 3.2.1. XRD Analysis of the Materials

**Figure 3:**
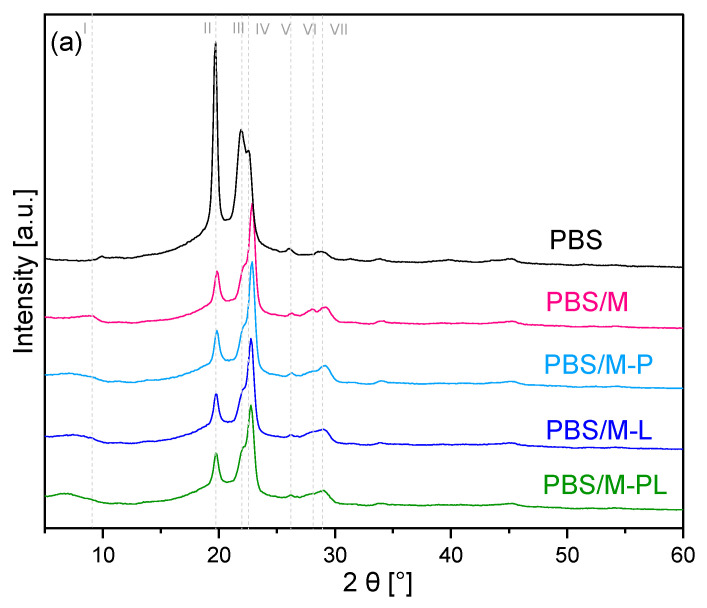

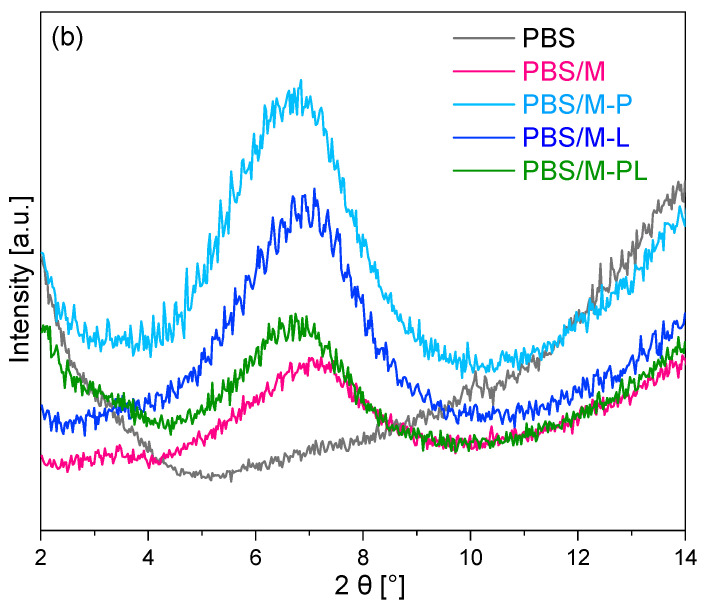
XRD patterns for PBS and its composites at full range (**a**), XRD patterns for PBS and its composites at a low value of 2θ (**b**).

**Figure 4:**
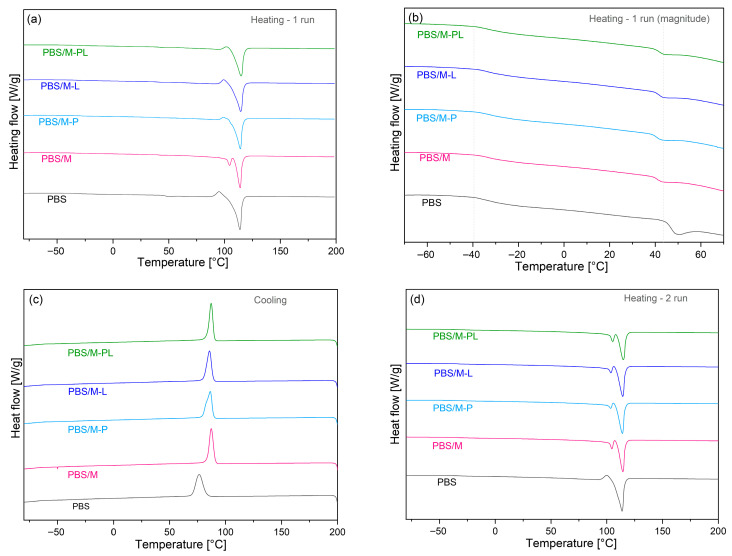
DSC thermograms for PBS and its composites. I heating (**a**), I heating (magnitude) (**b**), Cooling (**c**), II heating (**d**).

As seen in Figure 3a and Table 2, the addition of the filler slightly affected the PBS structure. The XRD patterns of PBS films exhibited diffraction peaks at 19.62, 21.92, 22.55, 26.12, 28.86, and 29.17°. The characteristic most pronounced peaks at 19.62, 21.92, 22.55, and 29.17° correspond to the diffractions from (020), (021) (110), and (111) planes, respectively [47,48]. Shifting of the two peaks with the highest intensity (PBS/M 19.62 and 22.55°) can be related to the shrinkage of the crystal lattice, probably caused by the occurrence of stacking faults interstitial or substitutional atoms and some residual stress in the absorbing layer, which can shift or broaden the peaks [49]. The peaks’ lower intensity can indicate the polymer’s intersection into the filler interlayer gallery [50] and the lower crystallinity of the PBS. The peaks marked with lines 1 and 6 came from the filler. Figure 4b shows peaks at lower 2θ values assigned to the montmorillonite interlayer gallery in the polymer matrix (Figure 3b, line 1). The low intensity of these peaks results from the filler’s “dilution” effect in the polymeric matrix [51]. Compared with the pure fillers, it can be seen that the interlayer space increased only in the case of pristine sodium aluminosilicate (from 1.20 to 1.25 nm), whereas the d_001_-spacing for all modified MMT decreased (Table 2). It can result from the “squeezing” of the platelets and migration of trapped terpenes into the polyester matrix. Similar results were also observed in other works related to polymer composites with OMMT. In work [52] d-spacing of MMT modified with dimeric surfactant and 4-decyloxyphenylacetamide shifted from 4.2° for their native form to 5.3° in the PBS matrix. The peak of MMT modified with cetyl trimethyl ammonium bromide (CTAB) indicates a high degree of intercalation (3.85°) after introduction into PBS shifted to 6.0° [53]. Bentonite modified with thymol and carvacrol that exhibited a d-spacing peak at 6.82° after addition into LDPE matrix shifted to 7.2° [54]. Moreover, in some works related to composites where two peaks for MMT can be observed, the first one with high intensity at low angle values related to the filler intercalation and broad but with low intensity at higher contact values (higher than pristine OMMT), indicating agglomeration of some fraction of introduced filler. This phenomenon was usually observed for the composites obtained via extrusion or injection molding, where higher temperature and high shear force were applied [48,55,56,57]. These changes can be caused by partial terpene evaporation induced by processing conditions. Lack of the shift towards low angle values indicates the formation of a conventional composite with the slight intercalated aluminosi-licate layers without their agglomeration, and not a nanocomposite [58].

#### 3.2.2. DSC Characterization: Phase Transitions

DSC analysis results from PBS and its composites are shown in Figure 4. During the first heating, melting peaks appeared as an effect of the thermal history of the materials connected with the melt processing. It can be seen that the introduction of the filler did not affect melting temperature (T_m_). However, the area of endothermic peaks (reflecting heat capacity) for the composites is slightly smaller, indicating lower crystallinity. For the second heating, there is a second T_m_ peak for PBS with unmodified MMT at ca. 104 °C that may be assigned to the formation of some crystals with lower thermal stability induced by the presence of the filler acting as a nucleating agent [58]. Crystallization during cooling occurred at higher temperatures in the case of the composites (Figure 4c). Below 50°, thermoformed PBS films exhibit two phase transitions after the first heating cycle and one after the second heating cycle (SI, Figure S3). A similar phenomenon was recorded in work [59].

The first change is a glass transition at T_g_ ca. −39 °C and is the same for all samples, whereas the second transition at 44.8 °C for unmodified PBS is shifted towards lower temperatures (38.3–39.5 °C) for the composites. At first glance, it can be interpreted as the second T_g._ However, this non-reversible peak with small enthalpy is assigned to the melting transition labelled as “annealing peak” for rigid-amorphous fraction (RAF) [60]. The slight shift towards lower temperature indicates that the presence of the filler in the matrix restricted the RAF chain interaction, and a weaker interaction between the polymer matrix and the filler was formed. Additionally, a test using DSC was carried out to determine the resistance of new materials to oxidation by determining the oxidation onset temperature (OOT). This allowed us to state that the composites are characterized by increased thermo-oxidative stability compared to pure PBS (SI, Table S1). The lowest recorded OOT values were for PBS (251 °C) and the highest value of 269 °C was for PBS/M-PL.

#### 3.2.3. Thermomechanical Behavior

**Figure 5:**
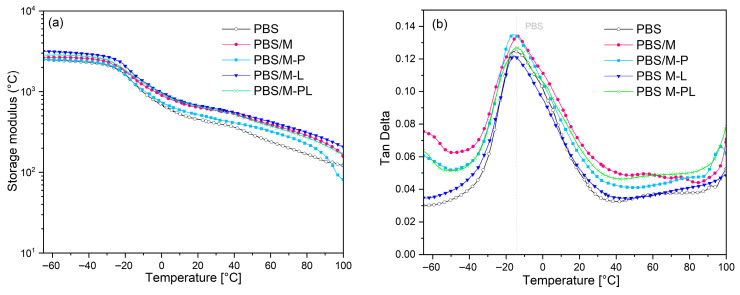
DMTA results: storage modulus, E′ (**a**); tanδ (**b**).

Dynamic thermal mechanical analysis was performed to study the viscoelastic properties of the composites. It can be noticed that storage modulus (E′) curves shown in Figure 5 for composites had higher values than those for pure PBS in the whole temperature range. An increase in E′ was also observed for PBS and PBSA films modified with OMMT [51,53,61]. It is related to the reinforcing effect of rigid structures of inorganic fillers dispersed in the polymeric matrix and improved stiffness of the composite material, compared to the reference PBS. The drop of E′ at ca. −26 °C is similar for all the samples, which corresponds with the DSC results (first T_g-DMTA_). The temperature of the maximum tanδ peak reflecting polymer chain movement for pure PBS (at ca. −9 °C) is shifted towards slightly lower temperatures for the composites, except for PBS/M and PBS/M-PL. This change can be an effect of the presence of terpene inside and on the surface of the filler and its intercalation. The exception for the sample containing the PL mixture can be related to the low content of the modifier in the hybrid filler. Usually, a simultaneous increase in the storage modulus with an increase in the relaxation temperature, interpreted as glass transition temperature, is interpreted as an effect indicating strong interactions between the filler and the polymeric matrix. Increased interactions between functional groups on the surface of the modified inorganic filler limit the mobility of polymer macromolecules in the interfacial region [62]. Based on the DMTA results, two separate mechanisms can be found among the analyzed materials. In the case of the PBS/M-P series, reduced values of both T_g-DMTA_ and E′were noted, which may be related to the more considerable amount of the non-bounded MMT low-molecular-weight modifier, which, after being dispersed in the polymeric matrix, showed a partial compatibilizing effect on PBS. This phenomenon can be related to the highest content of terpene in the modifier (Table 1). The remaining series were characterized by increased polymer–filler adhesion, further confirmed by the reduced tanδ value in the glass transition range compared to the PBS/M sample.

#### 3.2.4. Results of the Rheological Studies

**Figure 6:**
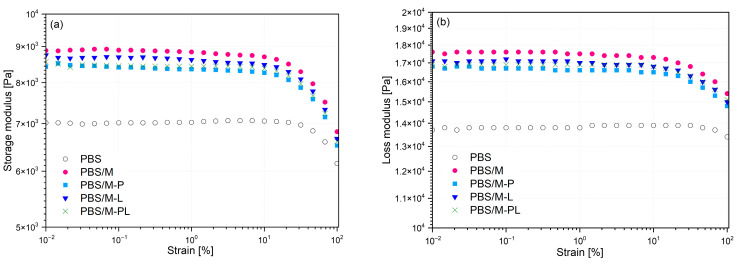
Oscillatory rheological results of strain-sweep experiments: Storage modulus (**a**), Loss modulus (**b**) conducted for PBS and its composites.

The results of rheological measurements performed in the strain-sweep experiment mode in the form of changes in storage (G′) and loss (G″) modulus as a function of strain are provided in Figure 6. For all series, the G″ values were higher than G′, which confirms the dominant viscous behavior of the molten PBS composites. The introduction of all types of fillers reduced the linear viscoelasticity (LVE) range observed during the strain-sweep experiment. This is an expected effect of the limitation of macromolecule mobility caused by interactions between the polymer and the dispersed filler in the matrix or the formation of clusters of the rigid filler particles in the polymer melt [63].

**Figure 7:**
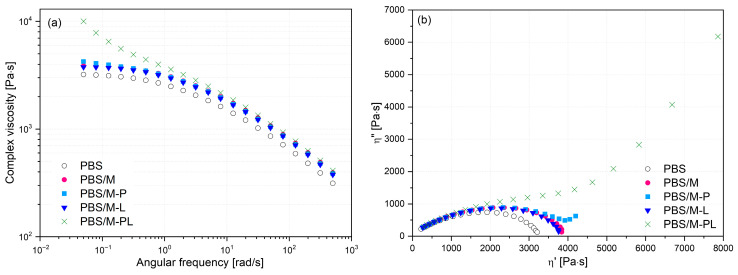
Oscillatory rheological results of frequency-sweep experiments realized for PBS and its composites: Complex viscosity (**a**), Cole-Cole plot (**b**).

In the case of angular frequency-sweep experiments (Figure 7), it can be observed that the introduction of M, M-P, and M-L did not change the nature of the complex viscosity curves compared to unmodified PBS (Figure 7a). This probably indicates the good dispersion of the filler or the development of agglomerated micrometric structures of the nano-sized fillers dispersed in the polymeric matrix. These curves show a Newtonian plateau in the range of small angular frequencies. The complex viscosity curve of PBS/M-PL is characterized by an increase in viscosity in the range of small angular frequencies, which is a characteristic effect for systems containing rigid filler structures forming a 3D-hindered network of non-melting domains remaining in physical contact, partially cross-linked or highly branched polymers [64,65,66]. The lack of dependence between G′ and angular frequency (ω) observed on the G′ (ω) curve in the range up to 0.1 rad/s for PBS/M-PL, may be interpreted as confirmation of reaching the rheological threshold of percolation by the filler in the polymer PBS/M-PL. Those data align with the previously mentioned possible intercalation of modified MMT by the polymer. Comparison of rheological oscillatory data results in the form of Cole–Cole plots, i.e., changes in η″ in the η′ function allows for a qualitative assessment of the miscibility or compatibility of the polymeric blends and composites [66,67]. The material is assumed to be compatible or miscible when the curves are arranged as smooth semicircle shapes (Figure 7b) [66]. With an increasing deviation from the model curve, which considers the flattening of the curves in the range of high viscosity values, the results indicate the occurrence of chemical interactions (in the case of partially cross-linked polymers) or the presence of physical interactions in the polymer bulk. In the considered case, only PBS/M-PL shows limited miscibility of the system resulting from physical interactions between the agglomerated filler particles or/and strong interfacial bonding in the polymer–filler interface.

#### 3.2.5. TGA Results

**Figure 8:**
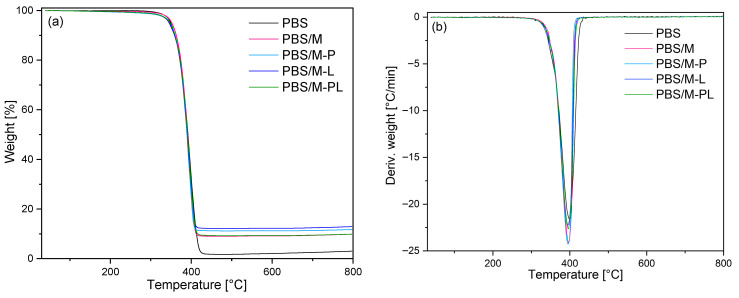
TGA (**a**) and DTG (**b**) curves of PBS and its composite films.

Analyzing TGA (Figure 8) results, it can be seen that the filler addition and its type did not affect the thermal stability of PBS. It also confirmed that PBS is not as sensitive to the chain-scission induced by terpenes as it was reported for PLA [50,61]. At a temperature range from 30 to 300 °C, there is no significant difference in the weight loss, indicating terpene volatility was lowered by trapping them in the aluminosilicate gallery. The highest weight loss at 270 °C, Figure S4, in SI was recorded for PBS/M-P, and this may be related to the adsorption of some fraction of pinene on the surface of MMT, not in the interlayer gallery.

#### 3.2.6. SEM Analysis

**Figure 9:**
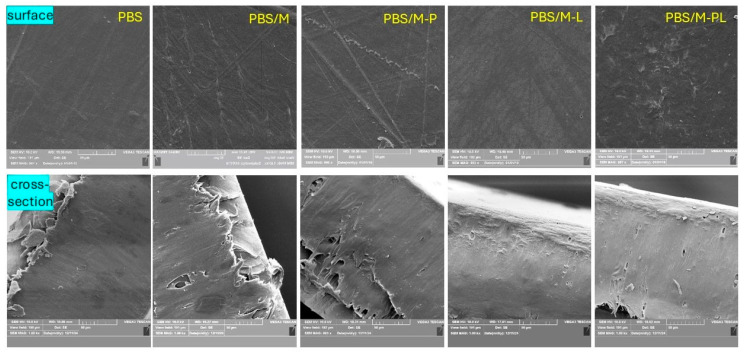
SEM graphs for film surface and cross-section (magnitude ×1000).

Microscopic analysis was performed to examine the surface of PBS films and PBS composites (Figure 9). Thermoformed PBS is much smoother than PBS film obtained via casting from the solvent [68]. It was observed that PBS and PPS/M-L films exhibited the smoothest surface among the samples. The other biocomposites were still homogenous but less smooth than neat PBS. It was caused by good filler distribution during the extrusion process, where filler particles/platelets are embedded in the matrix [69]. Similar results were observed in our previous study [32] or other works [49,70,71]. It is worth highlighting that good dispersion was reached for high content (10 wt%) of the filler, whereas in the mentioned works, the filler content was much lower. Different morphology with a quite rough surface was noticed for PBS modified with M-PL. It may be concluded that an agglomeration of additives was formed in the polyester matrix.

#### 3.2.7. Mechanical Properties

Table 3 shows the results of the mechanical tests, mainly Young’s modulus (YM), tensile strength (TS), and elongation at break (EB). It can be noticed that composites with pristine montmorillonite, as well as those modified with limonene and its mixture with pinene, have slightly higher YM and TS values and lower EB that indicates the formation of strong interaction of the fillers with the polymer chains that inhibit their mobility. As shown in previous studies, the changes in Young’s modulus determined in the static tensile test can be correlated with the storage modulus data determined by DMTA [72]. In the considered case, these results show a similar trend between both applied research methods. The reduction in EB observed in all cases results from introducing a separate phase into the polymer, constituting a point of stress accumulation during load [73,74]. The drop of the EB value and increase in YM and TS are also affected by the intercalation of the additive in the PBS matrix. In the case of clay modified with limonene, there was no improvement in TS, which can indicate that limonene is less compatible with the PBS than pinene and forms weaker physical bonding that does not restrict chain mobility. This can also be found in DMTA results where lower G′ and higher intensity of tanδ peak for PBS modified with MMT containing pinene were observed. Comparing the filler types, the most significant improvement of mechanical properties was obtained for PBS with the unmodified MMT (increase in TS and the lowest drop of EB values).

#### 3.2.8. Barrier Properties

Table 4 presents OTR values. Good oxygen-barrier properties of plastic materials are mainly required in food packaging, especially for products sensitive to oxidation [32]. Thermocompressed PBS film exhibited lower oxygen permeability (ca. 54 cm^3^/m^2^∙24 h) than cast-extruded film [75]. The value for PBS was slightly higher than poly(ethylene terephthalate) (PET) [76] and higher than poly(ethylene furanoate) (PEF) [77], but much lower than bio-based polyester-polylactide (85 cm^3^/m^2^∙24 h) [77] and slightly lower than PET-G [78], comparing thermocompressed materials in RH0%. The sequence of polyester packaging materials is as follows: PEF > PET > PBS > PET-G > PLA. The parameter values for the biocomposite are lower (38 cm^3^/m^2^∙24 h) than those for pure PBS film and the filler type did not significantly affect the sample oxygen permeability. The crystalline phases (XRD, Figure 3a), which formed an obstacle to gas diffusion, affected low OTR values [50]. The filler presence in the polymeric matrix limited gas transfer through the sample, creating a barrier to gas migration through the material. Similar results were observed for PBS films with sodium and organophilized MMT (Cloisite 30B) obtained via compression molding [48]. Adding the clay modified with clove essential oil into PLA/PBAT/TPS blend led to lower oxygen permeability than materials modified only using the essential oil [56].

The presence of the filler in the polymeric structure significantly decreased the barrier properties towards water vapor. In the case of PBS/M, WVTR reached ca. 10 g/m^2^∙24 h and was almost twice as low as PBS. Such a distinct drop in WVTR can result from the high content of the filler in the matrix, and similarly, as in the case of oxygen, forming a torus path for gas transmission [48,79,80]. Moreover, sodium montmorillonite can exhibit better compatibility with PBS matrix than with PE. Giannakas [46] showed that the addition of M or M modified with EOs, led to higher WVTR in LDPE composites. The values for the biocomposites are comparable to commercially used PET [81] known as a high barrier material among the most popular packaging plastics.

#### 3.2.9. Wettability—Surface Contact Angle

Water contact angle values (WCA) of the material’s surface, reflecting their wettability and hydrophilic/hydrophobic character, are listed in Table 4. WCA for unmodified PBS film (67°) obtained via thermo-compression is similar to that of PBS foil formed via cast extrusion [75] and higher than that of PBS film formed via organic solvent casting (55°) [66]. The difference is related to the smooth morphology (Figure 9) of the samples presented in this work. The introduction of the fillers statistically significantly increased the hydrophobic character of the biocomposites. The highest values (77°) were obtained for samples with M modified with pinene. It can result from the character of the modifier and its content in the polymer matrix (Table 1).

#### 3.2.10. Antiviral Properties

**Figure 10:**
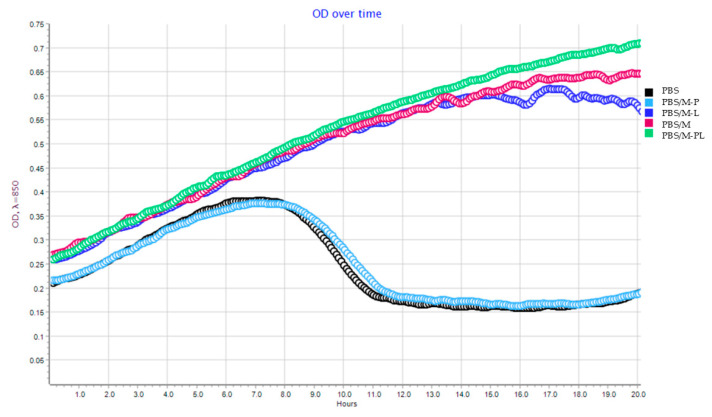
The optical density (OD) over time of the *P. syringe* with Φ6 particles after its cultivation/incubation with PBS and its composite films. Addition of phages when OD—0.2; amount of phage MOI—1.

The outcomes of the antiviral evaluations showed that an OD fall was observed after 8 h of incubation of bacterial host *P. syringae* with the Φ6 phage cultivated with the PBS film (control sample) and/or with its composite PBS/M-P, confirming that these two samples did not influence the phage particles (Figure 10).

The lack of antiviral activity of these two films was confirmed by an experiment which was performed according to a modified ISO 22196-2011 standard [36]. The results of the experiment (Figure S5) showed that the titers of the Φ6 lysates after their incubation with the PBS film (control sample) and/or with its composite PBS/M-P were 1.29 × 10^7^ and 1.27 × 10^7^ PFU/mL, respectively. Statistical analysis confirmed that the differences were not significant. On the contrary, the OD fall for the composites: PBS/M-PL, PBS/M-L, and PBS/M, was not observed after 8 h of incubation, but a slight OD fall was observed for PBS/M-L after 20 h cultivation. These results confirmed that PBS/M, PBS/M-L, and PBS/M-PL composites were very effective against Φ6 phage. The effectiveness of the described PBS composites was also confirmed by a test performed according to a modified ISO 22196-2011 standard [36], showing that the titers of Φ6 lysates (Figure S5) were reduced to zero. Statistical analysis confirmed that the differences were significant. However, considering that PBS/M-PL was slightly more active than PBS/M-L film and that PBS/M-P was inactive towards bacteriophage, it may be concluded that synergistic effects between limonene and pinene were noted. Similar results were demonstrated in the previous study [82], which confirmed that polyethylene (PE) films containing the mixture of scCO_2_ extracts of rosemary, raspberry, and pomegranate fruit in the PE matrix exhibited high activity against Φ6 phage. However, the other work [32] determined that biopolymer resin based on PHB/PLA blend modified with *Hypericum* L., *Urtica* L., and *Chelidonium* L. extracts mixture and zinc oxide nanoparticles hybridized with the extracts mixture showed moderate activity against Φ6 phage. Antiviral activity of polymer/biopolymer films can be achieved not only by introducing active substances into the polymer matrix, but also by covering the films with an active coating based on a biopolymer coating carrier. The results of the previous work [83] demonstrated that the coatings containing zinc oxide (ZnO) nanoparticles and *F. betulina*, *Verbascum* L., or *U. tomentosa* extracts caused a complete reduction in the bacteriophage titer compared to the control sample (non-coated film). As an additional confirmation of the results obtained according to the ISO 22196-2011 standard [36] (including the double agar layer technique), supplementary series of tests were performed. They were based on observing the OD (optical density) of the host culture during co-incubation with bacteriophages that had been previously exposed to the active or non-active films. The analysis of the host’s growth rate in real-time showed a clear OD fall after 11 h of co-incubation with Φ6 particles previously incubated with the non-active film, proving that the film did not exert antiviral activity, as the phage remained viable and infective. However, in the case of films coated with the active layers, an OD fall was not observed even after 23 h of phage cultivation with the host, meaning that the Φ6 phage particles had been rendered inactive. These two methods confirmed that the coatings had antiviral properties. Similar results were noted in the work [84] describing the coating modified with chokeberry extract mixed with zinc oxide particles. An OD fall was not noted, and the reduction in the bacteriophage titer compared to the control sample was not noticed, meaning that bacteriophage particles were present. Contrary observations were found for the coatings with CO_2_ raspberry seed extract and ZnO or zinc stearate particles. The differences between phage titers were not significant and an OD fall was observed after 7 h of the cultivation of the *P. syringae* with the Φ6 particles, despite their previous incubation with PLA film or PLA film covered with the two coatings mentioned above. Both methods were used to confirm the antiviral properties of the coating analyzed in the previous work [85] and demonstrated that films with the coatings enriched with tea tree oil were highly active against the Φ6 phage.

As emphasized in the graph (Figure 10), the PBS/M composite impacted the bacteriophage effectiveness. It can suggest that the antiviral activity might be related to the increasing adhesion of phage on the surface with adhesive properties that could be added into a polymer matrix instead of active/antiviral substances. It corresponds to SEM analysis, which revealed a slightly more developed surface of PBS/M films than the other series (Figure 9). Orchibat E. et al. [86] confirmed that the adhesion of bacteriophages on the surface is possible for 12 different industrial plastics (acrylonitrile butadiene styrene, high-impact polystyrene, poly-ε-caproamide, polycarbonate, polyethylene, PET, poly(methyl methacrylate), polypropylene, polystyrene, polytetrafluoroethylene, polyurethane, and polyvinyl chloride). The authors selected and tested T4, MS2, and M13 as examples of bacteriophages. T4 was selected as a representative of a dsDNA-tail possessing phages. The MS2 bacteriophage was chosen as an ssRNA phage that has an icosahedral structure, and which frequently serves as a surrogate for eukaryotic viruses. All three studied phages share a host—*Escherichia coli*. The Φ6 phage, which has a lipid envelope, was selected for the current experiments. The results of the tests showed that unlike PBS, PBS/M made the adhesion of phi6 on its surface possible, confirming that the modification of the polymer might facilitate this process. Richter et al. [87] mentioned that hydrophobicity is one of the most critical factors governing the adsorption of molecules and objects, such as virions, on surfaces. Even a moderate change in the wetting angle of plastic surfaces causes a drastic increase in the viruses (phages) retained on their surfaces due to adsorption. The authors showed that the polypropylene surface of the Eppendorf vial-type and Falcon vial-type test tubes could accommodate from around 10^8^ PFU/mL to around 10^10^ PFU/mL of virions from the suspension. They confirmed that the adsorption of phage particles onto the containers’ walls might have resulted in complete scavenging of virions from the bulk. The authors also discovered that the phage adsorption on polymer surfaces occurred similarly in T4 and MS2 phages, despite differences in the phages’ physical structure. It may be concluded that the adhesion of phi6 phage would be similar.

It is worth mentioning that Φ6 bacteriophage may be considered a SARS-CoV-2 surrogate because it is enveloped by a lipid external layer as well [88,89,90]. Summarizing these outcomes, it can be assumed that PBS/M, PBS/M-L, and PBS/M-PL films that were very effective against Φ6 particles might also lead to the decrease in the number of SARS-CoV-2 particles and, as a result, limit the spread of coronavirus when it is needed.

## 4. Summary

The work aimed to prepare functional biocomposites based on poly(butylene succinate). Selected monoterpenes, pinene, limonene, or their mixture, effectively modified sodium montmorillonite clay used as a filler. XRD confirmed the intercalation of the modifier in the filler gallery. Moreover, the application of natural-based modifiers caused changes in the structure of composites in the interfacial polymer–filler interactions. This allowed for the improvement of the strength properties of composites compared to unmodified PBS. Introducing the filler into PBS slightly increased tensile strength, but the barrier properties towards gases (oxygen and water vapor) were significantly improved. WVTR values for composites were almost twice as low as those for pure PBS. FTIR, XRD, and DMTA revealed that the introduction of the additive did not significantly affect the morphology, and the filler remained intercalated and well dispersed in the matrix (SEM results).

The PBS/M, PBS/M-PL, and PBS/M-L composites were confirmed to be very effective against Φ6 phage, and the effectiveness of the PBS/M-PL was the highest. The functional biocomposites can find an application as safe medical materials and food packaging, restricting the spread of virus particles, e.g., by human hands. The current work, for the first time, showed preliminary studies on bio-based and biodegradable poly(butylene succinate) modified with montmorillonite modified with terpene. Due to the rather low content (according to TGA) of the active modifiers, the antimicrobial properties towards different pathogenic microorganisms are limited. Thus, further studies are required to develop active materials with wider spectra of activity towards different bacterial strains. Due to partial entrapping of the terpenes in the clay platelets, their release from the polymer can be gradual and this issue is worth investigating in the future.

## Figures and Tables

**Table 1:** Modifier content in the clay.

Filler	Modifier Type	Modifier Content
M-P	P (pinene)	13.8%
M-L	L (limonene)	6.6%
M-PL	PL (pinene + limonene)	8.8%

**Table 2:** 2theta values for PBS and its composites.

Sample	Peaks Assigned to MMT (d-Spacing)		Peaks Assigned to PBS Matrix				
Line (Figure 3a)	I (001)	VI (005)	II (020)	III (021)	IV (110)	V (121)	VII (111)
PBS	-	-	19.62°	21.92°	22.55°	26.1°	29.17°
PBS/M	7.05° (1.25 nm)	28.01°	19.82°	-	22.97°	-	29.07°
PBS/M-P	6.79° (1.30 nm)	28.01°	19.82°	-	22.76°	-	29.07°
PBS/M-L	6.72° (1.32 nm)	28.01°	19.82°	-	22.76°	-	29.07°
PBS/M-PL	6.75° (1.31 nm)	28.01°	19.82°	-	22.76°	-	29.07°

**Table 3:** The mechanical test results.

Sample	Young’s Modulus [MPa]	Tensile Strength [MPa]	Elongation at Break [%]
PBS	473 (±34) ^c^	28 (±1.5) ^a,b^	18 (±5.0) ^a^
PBS/M	511 (±72) ^c^	34 (±2.8) ^a^	11 (±1.2) ^b^
PBS/M-P	718 (±72) ^a,b^	30 (±3.4) ^a,b^	8 (±1.4) ^b^
PBS M-L	684 (±42) ^b^	27 (±3.6) ^b^	8 (±1.1) ^b^
PBS/M-PL	625 (±34) ^b^	32 (±3.3) ^a,b^	10 (±1.4) ^b^

^a–c^—averages marked with the same letters do not differ significantly from each other for *p* < 0.05.

**Table 4:** OTR, WVTR, and water contact angle (WCA) values.

Sample	Barrier Properties			WCA [°]
	OTR _RH 0%_ [cm^3^/m^2^∙24 h]	WVTR_RH 100%_ [g/m^2^∙24 h]	WVTR_RH 90%_ * [g/m^2^/24 h]	
PBS	52 (±3.2)	21 (±1.1)	19 (±1.0)	67 (±2.7) ^d^
PBS/M	39 (±3.4)	11 (±0.2)	10 (±0.1)	73 (±2.8) ^b,c^
PBS/M-P	38 (±7.5)	13 (±1.4)	12 (±1.3)	77 (±0.9) ^a,b^
PBS M-L	38 (±1.6)	12 (±1.9)	11 (±1.7)	72 (±1.2) ^c^
PBS/M-PL	42 (±3.7)	13 (±0.2)	12 (±0.1)	77 (±2.0) ^a^

* compensated; ^a–d^—averages marked with the same letters do not differ significantly from each other for *p* < 0.05.

## Data Availability

The original contributions presented in this study are included in the article. Further inquiries can be directed to the corresponding author.

## Supplementary Materials

The following supporting information can be downloaded at: https://www.mdpi.com/article/10.3390/polym17222984/s1, Figure S1: TGA curves of Na-MMT, terpenes, their mixture (PL), and modified MMT; Figure S2: FTIR spectra of PBS and biocomposite films; Figure S3: DSC curves for thermocompressed PBS films and its composites for the second heating cycle; Figure S4: TGA curve with magnitude of the weight drop; and Figure S5: The influence of PBS and its composites on Φ6 titer. One-way ANOVA: ****—*p* < 0.0001, ns—not significant; Table S1: OOT values (determined by DSC) and scavenging efficiency of DPPH radical.

## Abbreviations

The following abbreviations are used in this manuscript:

DSC	Diffraction scanning calorimetry
DMTA	Dynamic mechanical thermal analysis
FTIR	Fourier Transform Infrared Spectroscopy
L	Limonene
M	Montmorillonite
OTR	Oxygen transmission rate
WCA	Water contact angle
WVTR	Water vapor transmission rate
P	Pinene
PBS	Poly(butylene succinate)
SEM	Scanning Electron Microscopy
XRD	X-ray diffractometry
