# Supplementary material for: Physico-Chemical, Rheological, and Antiviral Properties of Poly(butylene succinate) Biocomposites with Terpene—Hydrophobized Montmorillonite
Source: Polymers (Basel). 2025 Nov 10;17(22):2984. doi: 10.3390/polym17222984 (PMC12656094; doi:10.3390/polym17222984)
Supplement: Supplementary file 1 [file polymers-17-02984-s001.zip › polymers-3968343-supplementary.pdf]

## SUPPLEMENTARY INFORMATION

# Physico-chemical, rheological and antiviral properties of poly(butylene succinate) biocomposites with terpene-hydrophobized montmorillonites

Magdalena Zdanowicz[1*], Mateusz Barczewski[2], Małgorzata Mizielińska[1], Piotr Miądlicki[3]

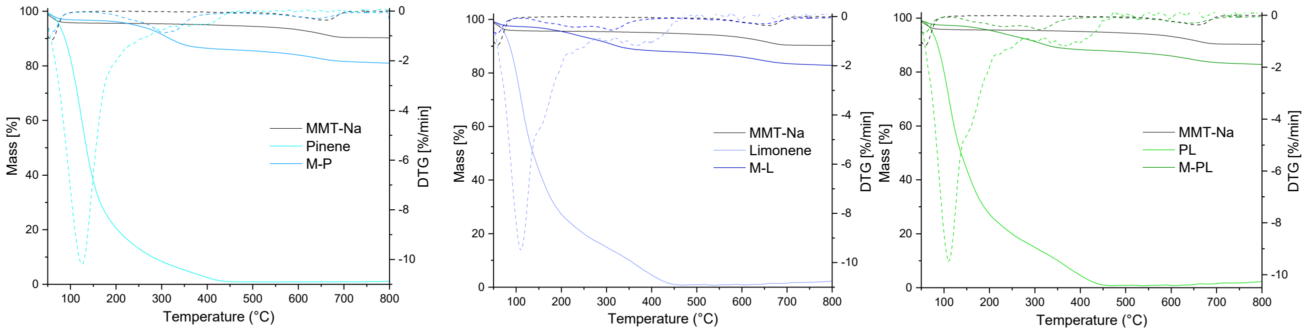


**Figure S1.** TGA curves of Na-MMT, terpenes, their mixture (PL) and modified MMT

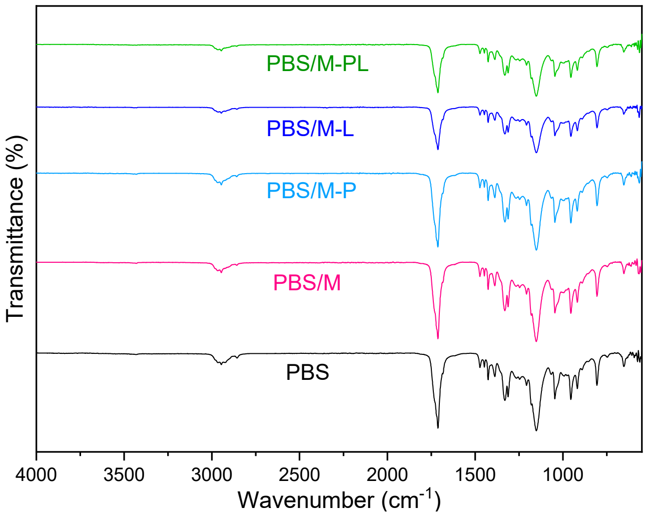


**Figure S2**. FTIR spectra of PBS and biocomposite films.

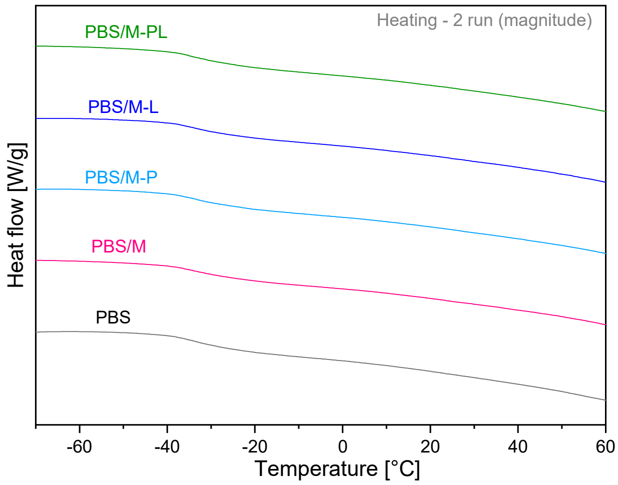


**Figure S3.** DSC curves for thermocompressed PBS films and its composites for second heating cycle.

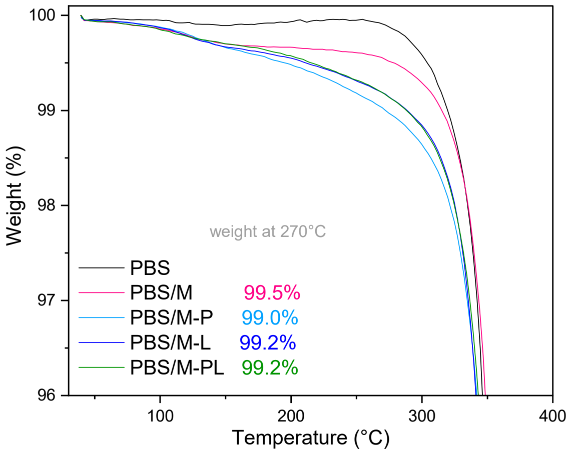


**Figure S4.** TGA curve with magnitude on the weight drop.

## Antioxidative properties - %DPPH

Thermocompressed samples were cut into 20 × 20 mm parts placed in falcons, 1 mL of distilled water was added, then 3 mL of methanol and 1 mL of methanolic solution of DPPH and then the samples were kept for 10 min in a dark place. The solution from the stored samples was measured immediately using a UV-Vis spectrophotometer (Evolution 220 UV-Visible Spectrophotometer, Thermo Scientific), at a wavelength of 517 nm. The degree of scavenged radicals was calculated according to the equation DPPH% = $(A_{DPPH} - A_s) \times 100\%/ADPPH$, where $A_{DPPH}$ is the absorbance of the control sample and $A_s$ is the absorbance of the studied liquid above the film. The results are listed in Table S1.

**Table S1**. OOT values (determined by DSC) and scavenging efficiency of DPPH radical

| Sample | OOT [°C] | %DPPH |
|---|---|---|
| PBS | 251.4 | 10.1 |
| PBS/M | 268.1 | 9.3 |
| PBS/M-P | 262.6 | 33.6 |
| PBS/M-L | 256.0 | 13.5 |
| PMS/M-PL | 268.5 | 10.4 |

Results of an investigation of free radical scavenging using DPPH reagent are listed in SI, Table S1. All samples exhibited very weak antioxidative properties, except for PBS/M-P, where the value was the highest (33.6%), thus only PBS/M-P had some potential as active food packaging. This can be related to the highest content (two times higher than L) of P in the filler (Table 1). LDPE films M and organophilized M with thyme, oregano and basil EOs exhibited higher %DPPPH [46].

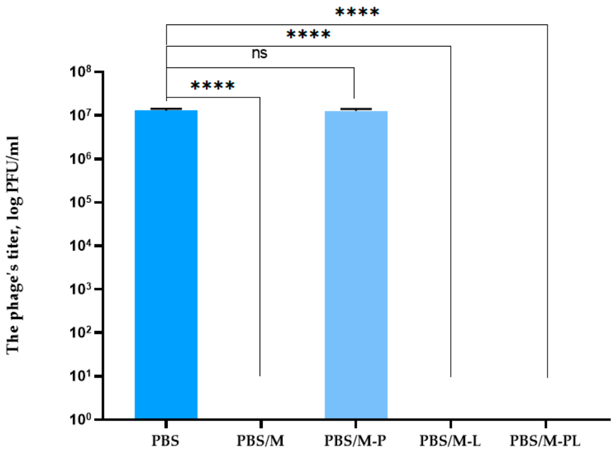


**Figure S5.** The influence of PBS and its composites on Φ6 titer. One-way ANOVA: ****—$p < 0.0001$, ns – not significant.
